# Soft drink intake is associated with weight gain, regardless of physical activity levels: the health workers cohort study

**DOI:** 10.1186/s12966-020-00963-2

**Published:** 2020-05-12

**Authors:** Romina González-Morales, Francisco Canto-Osorio, Dalia Stern, Luz María Sánchez-Romero, Leticia Torres-Ibarra, Rubí Hernández-López, Berenice Rivera-Paredez, Dèsirée Vidaña-Pérez, Paula Ramírez-Palacios, Jorge Salmerón, Barry M. Popkin, Tonatiuh Barrientos-Gutiérrez

**Affiliations:** 1 Center for Population Health Research, National Institute of Public Health, 62100 Cuernavaca, Mor. Mexico; 2 CONACyT- Center for Population Health Research, National Institute of Public Health, Cuernavaca, Mor. Mexico; 3 Center for Research in Policies, Population and Health, Faculty of Medicine, National Autonomous University of Mexico, Mexico city, Mexico; 4 Epidemiological Research and Health Services Unit, Mexican Institute of Social Security, Cuernavaca, Mor., Mexico; 5 Department of Nutrition, Gillings School of Global Public Health, University of North Carolina at Chapel Hill, Chapel Hill, North Carolina USA

**Keywords:** Soft drink intake, Weight, Physical activity

## Abstract

**Background:**

While soft drink intake is positively associated with weight gain, no previous study has investigated whether leisure-time physical activity modifies this association. We estimated the association between soft drink intake and body weight, and explored if this association differed by levels of leisure-time physical activity.

**Methods:**

We used data from the health workers cohort study, a prospective study of Mexican adults (20 to 85y old), including 1268 health workers and their families, who were assessed at baseline (2004–2006) and follow-up (2010–2012). We assessed soft drink intake (cola and flavored soda) using a validated food frequency questionnaire. We measured leisure-time physical activity using a self-report questionnaire, and categorized according to the 2010 World Health Organization (WHO) recommendations. Body weight was measured by trained personnel. The association between changes in soft drink intake and weight change, and if such association varied by levels of physical activity was estimated through fixed-effect models.

**Results:**

An increase in one serving per day of soft drink was associated with 0.10 kg (95% CI 0.00, 0.19) increase in weight per year. This association was not modified by leisure-time physical activity, as demonstrated by the magnitude of the coefficient of the interaction between soft drink, leisure-time physical activity, and time (− 0.03 kg, 95% CI − 0.27 to 0.21); people who complied with the WHO physical activity recommendations gained 0.36 kg/year per serving of soft drink, compared to 0.48 kg/year for people without sufficient physical activity.

**Conclusions:**

Soft drink intake was associated with weight gain. Leisure-time physical activity did not modify the association between soft drink intake and weight gain. This finding challenges the idea that leisure-time physical activity is sufficient to counterbalance weight gain associated to soft drink intake.

## Introduction

Sugar-sweetened beverages (SSB) intake is a major modifiable risk factor for overweight and obesity. Previous meta-analyses support an association between SSB intake and weight gain [1–3], with weight gains ranging from 0.12 to 0.22 kg/year for every serving (355 ml) consumed per day [3]. The causal pathways between SSB intake and obesity are clear, and involve an excess of calories with limited compensation, leading to overconsumption of energy [4, 5]. This is an emergent problem for countries with high SSB intake, like Mexico. While the global annual per-capita consumption was 57 L, in México, SSB intake was 167 L per person per year [6]. At the same time, the prevalence of overweight and obesity increased from 71.3% in 2012 to 75.2% in 2018 [7].

Experimental and observational studies have provided evidence of a link between SSB and obesity [8]. However, experimental studies have had short durations, ranging from 3 weeks to 6 months, providing a limited time frame for body weight change [3]. Observational studies have had longer durations, from 1 to 20 years [3]; yet, most studies have assumed that baseline SSB intake remains unchanged [2, 9–11], an assumption that could lead to biased estimates in dietary studies [12]. A subgroup of observational studies has used fixed effect models to estimate the impact of changes in intake over changes in weight, capturing longer time frames while maintaining a tight control for time invariant confounders [13]. Fixed effect estimates are scarce and have been mostly limited to high-income countries, with little information from other contexts. Having better estimates of weight change associated with changes in SSB intake from low and middle income countries is key to improve the global estimates of the impact of SSB intake on obesity [14–18].

The role of physical activity in the association between SSB intake and weight gain has been controversial and requires further analysis. Most studies that have analyzed the association between SSB intake and body weight have adjusted for physical activity, assuming that physical activity is a confounder of the association between SSB and weight (see diagram “a” from Fig. S1, available in the Additional file 1) [1, 19, 20]. However, a key point of discussion is the potential role that physical activity could play to counter the weight gain produced by SSB intake. Research funded by the SSB industry assumes that leisure-time physical activity will offset the positive energy imbalance produced by SSB intake [21, 22]. This implies that given the same level of exposure to SSB intake, people who exercise would gain less weight than those who do not exercise, making physical activity an effect modifier of the SSB and body weight association (see diagram “b” from Fig. S1, available in the Additional file 1). To our knowledge, no study has specifically tested this hypothesis. This study had two aims, first we estimated the change in body weight associated to a change in soft drink intake over 6 years, considering physical activity as a confounder. Secondly, we determined if the soft drink-weight change association was modified by leisure time physical activity. Using a prospective cohort study including adult males and females, with objective body weight measurements, we estimated the association of SSB changes in intake and body weight change, for the whole cohort, and by different levels of leisure-physical activity.

## Methods

### Study design and population

We used data from the health workers cohort study (HWCS), a longitudinal study of Mexican adults established in 2004–2006 to evaluate lifestyle characteristics and their association with chronic diseases. The source population included Mexican adult healthcare personnel and their relatives (20 to 85y old) from three institutions: the Mexican Social Security Institute and the National Institute of Public Health, both located in Cuernavaca, Morelos, and the Autonomous University of the State of Mexico located in Toluca, Mexico. At baseline, 10,729 subjects were enrolled. However, due to budget restrictions, only employees from the Mexican Social Security Institute and their families (*n* = 2500) were invited to participate in the second wave of data collection (2010–2012), from whom 1923 answered the follow-up questionnaire (76.9% follow-up rate within the Mexican Social Security Institute). Participants responded to self-administered questionnaires about sociodemographic characteristics, diet, lifestyle, and medical conditions. Clinical and anthropometric measurements were obtained by trained personnel by appointments at a health clinic. Detailed information about the HWCS and the questionnaires used for data collection have been described previously.(4).

For the present analysis, we used data from the two waves. We excluded participants < 19 years old (*n* = 150), those with missing weight (*n* = 156), soft drink intake (*n* = 143), or leisure-time physical activity (*n* = 5), pregnant women (*n* = 5), participants with implausible caloric intakes (< 600 or > 6000 kcal/day, *n* = 135, [23]) and those with missing data on covariates (*n* = 61). The final analytical sample included 1268 participants (see Fig. S2, Additional File 1).

### Assessment of soft drink intake

Dietary intake was self-reported at each wave using a previously validated 116-item semi-quantitative food frequency questionnaire (FFQ) [24]. Each item asked participants to specify, on average, how often they had consumed a common unit or portion size of the food or beverage over the previous year. Ten multiple-choice frequencies of intake were possible: ≥6 per day, 4–5 per day, 2–3 per day, 1 per day, 5–6 per week, 2–4 per week, 1 per week, 1–3 per month, ≤1 per month, and never. The FFQ included two items on the intake of soft drink (cola and flavored sodas). Diet sodas were not included in this analysis. We converted frequency responses of soft drink to servings per day (predefined portion size of 355 mL) [24].

### Assessment of anthropometric measures

Trained nurses measured body weight using a calibrated electronic scale (Tanita, model BC-533) to the nearest 0.1 kg (0.1–130 kg range) in both waves. Participants were measured at each wave wearing minimum clothing. Trained nurses measured height with a stadiometer (Seca) to the nearest 0.1 cm, with participants standing barefoot. Body mass index (BMI) was calculated using Quetelet’s calculation [25]: weight (kilograms) divided by height (meters squared).

### Assessment of leisure-time physical activity

Leisure-time physical activity was measured in both waves using a previously validated self-reported questionnaire [26, 27], which collected information on the frequency (days/week), time (hours/week), and intensity (light, moderate and vigorous) of 14 activities during a typical week over the last year. This questionnaire has been validated with an accelerometer in a Spanish-speaking population (Spearman correlation coefficient of 0.51 (95% CI 0.23 to 0.71)) [27]. We calculated the number of minutes per day of leisure-time physical activity based on the self-reported amount of time (min/week) spent walking, running, cycling, jogging and playing other sports. Following the WHO guidelines, we categorized participants leisure-time physical activity as low (< 21.44 min/day, which is equal to < 150 min/week of moderate to vigorous activity), or high (≥21.44 min /day, which is equal to ≥150 min/week of moderate to vigorous activity) [28]. We also created a continuous variable for minutes/day of leisure-time physical activity. Further, following the Physical Activities Guidelines for Americans, we categorized minutes of metabolic equivalent units (METs) of leisure-time physical activity in minutes per day as low (< 71.5 METs minutes/day, which is equal to < 500 METs minutes/week of moderate to vigorous activity) or high (≥71.5 METs minutes/day, which is equal to ≥500 METs minutes/week of moderate to vigorous activity).

### Assessment of covariates

The 2004–2006 and 2010–2012 questionnaires, asked participants to self-report their age, sex, education, lifestyle habits such as smoking, sleeping, sedentary behaviors, alcohol intake, and any recent physician-diagnosed disease. Based on previous reports on dietary associations with weight change [15], we created nine food groups and alcohol intake using data from the FFQ. Multiple-choice frequencies of intake were converted to servings per day for food groups, (5) and grams per day for alcohol intake. We included the following food groups: red meat, vegetables, fruits, total dairy, nuts, yogurt, white bread, tortillas, and orange juice [24]. Participants self-reported the amount of time spent watching television and using a computer. These two variables were used to create a screen-time use variable in hours per week, as a proxy for sedentary behavior. Sleep duration was also self-reported as hours per day. We created a variable to identify participants with a self-reported medical diagnosis of chronic diseases that could affect soft drink intake and body weight, such as cirrhosis, diabetes, cardiovascular disease, cancer, or kidney disease. Finally, we created a time variable in years to take into consideration the time elapsed from the date of response of the first questionnaire to the date of return of the second questionnaire.

### Statistical analysis

For descriptive analyses, we estimated the means and standard deviation (SD) for continuous variables and frequencies for categorical variables at baseline and follow-up; we also calculated the average change at the population level between baseline and follow-up. To estimate the association between soft drink intake and weight change over time, we used a fixed-effect model. This model removes all the time-invariant observed and unobserved characteristics related to soft drink intake and weight. Yet, to allow for a more flexible use of time [29], and to take into account different trajectories of weight over time, we included two-way interaction terms between: time (continuous, years) and soft drink intake (continuous, servings/day), time and sex (male/female), time and age at baseline (continuous, years) and time and leisure-time physical activity (categorical: low (< 21.44 min /day) and high (≥21.44 min /day)). Age and sex were centered at the baseline mean. In addition, we adjusted for time-varying covariates that have been previously identified as risk factors for weight gain, and that are also associated with soft drink intake: education (elementary school, secondary or high school, and college or higher), smoking (never, past, current) [30], screen time (3–4 h/week, 5–6 h/week, > 7 h/week) [31], sleep (continuous) [31], alcohol intake (low: δ12.5 g/day, and high: > 12.5 g/day) [32], chronic diseases (yes/no), and food groups (all continuous as servings/day) (Model 1).

To test the hypothesis that leisure-time physical activity modifies the association between soft drink intake and body weight over time, we included a three-way interaction term between soft drink, time, and leisure-time physical activity (Model 2). This model also included two-way interaction terms for soft drink and time, soft drink and leisure-time physical activity, leisure-time physical activity and time, sex and time, and age at baseline and time. Model 2 was also adjusted for the time-varying covariates included in model 1. In both models, we used robust standard error estimators to correct for the non-independence of participants that belonged to the same family. To aid with interpretation, we used model 2 coefficients to predict the average marginal weight change per year at pre-specified units of soft drink intake (zero, one, and two servings per day) and by levels of leisure-time physical activity. We do not present predictions for weight change for three or more servings because that group comprised only 3% of the sample. Details about the modeling strategy are included in the Additional File 2. To evaluate the goodness of fit, we used the Akaike Information Criterion (AIC) and Bayesian Information Criterion (BIC).

Finally, we conducted sensitivity analyses to evaluate the robustness of these findings. First, we replaced the dichotomous leisure-time physical activity variable for a continuous variable (minutes/day). We estimated the predicted average weight change values for pre-specified units of soft drink intake (zero, one, and two servings per day) and by percentiles of leisure-time physical activity: 1st (0 min/day), 25th (3.21 min/day), 50th (12.86 min/day), 75th (31.35 min/day) and 90th (60 min/day). Secondly, we substituted the dichotomous leisure-time physical activity variable of minutes per day for METs per day. Third, to explore the potential influence of baseline weight on these results, we stratified the models by baseline BMI categories. Although physical activity in minutes per day is the most conventional measure, we used METs to have a more robust measure of time and intensity of physical activity. All analyses were performed using STATA 14.2 [33].

## Results

At baseline, 74.2% of participants were women and had a mean age of 45.3 years old (SD ± 12.7, range 19 to 82 yr). Average soft drink intake at baseline was 0.5 servings/day (SD ± 0.7) and 0.4 servings/day (SD ± 0.6) at follow-up. The average weight increased 1.1 kg between 2004 and 2010. Mean leisure-time physical activity (min/day) was 24.10 (SD ± 29.19) at baseline and 21.28 (SD ± 26.72) at follow-up. The prevalence of overweight and obesity increased by 4.4% from 2004 to 2010. In addition, the prevalence of performing low levels of leisure-time physical activity also increased by 5.3% between 2004 and 2010. In the same time period, the percentage of participants in the category of ≥7 h of screen-time increased from 19.2% to 24.5%. The percentage of participants with chronic diseases increased from 9.4% in 2004 to 14.8% in 2010 (Table 1).

**Table 1:** Descriptive characteristics of the health workers cohort study (HWCS) sample at baseline (2004) and follow up (2010) (*N* = 1268)

Characteristics	2004 Mean (SD)	2010 Mean (SD)	Average change Mean difference (SD)
Age (years)	45.3 (12.7)	52.3 (12.8)	7.0 (1.5)
Soft drink intake (servings/ day)	0.5 (0.7)	0.4 (0.6)	−0.1 (0.6)
Weight (Kg)	66.9 (13.1)	68.0 (13.7)	1.1 (5.7)
BMI (kg/m^2^)	26.5 (4.3)	27.1 (4.5)	0.6 (2.2)
BMI category, %			
< 25 kg/m^2^	38.9	35.5	−3.4
≥ 25 kg/m^2^	60.1	64.5	4.4
Physical activity (min/day)	24.1 (29.2)	21.3 (26.7)	−2.8 (33.6)
Physical activity (min/day), %			
Low (< 21.44 min/day)	61.3	66.6	5.3
High (≥21.44 min/day)	38.7	33.4	−5.3
Education, %			
Elementary school	12.4	12.2	−0.2
Secondary or high school	37.4	36.7	−0.7
College or higher	50.2	51.1	0.9
Smoking status, %			
Never	58.3	55.5	−2.8
Past	24.8	32.1	7.3
Current	17.0	12.3	−4.7
Chronic diseases, %			
No	90.6	85.2	−5.4
Yes	9.4	14.8	5.4
Screen-time/ week, %			
3–4 h	59.4	60.2	0.8
5–6 h	21.4	15.3	−6.1
≥ 7 h	19.2	24.5	5.3
Total daily sleep (hours)	8.0 (1.8)	7.7 (1.8)	−0.3 (2.1)
Alcohol intake (grams/day), %			
Low (≤12.5 g/day)	91.4	93.5	2.1
High (> 12.5 g/day)	8.6	6.5	−2.1
Food groups (servings/ day)			
Red meat (servings/day)	0.9 (0.71)	0.5 (0.5)	−0.4 (0.8)
Fruits (servings/day)	4.0 (4.3)	4.2 (4.6)	0.2 (5.5)
Vegetables (servings/day)	4.3 (3.0)	4.3 (5.0)	−0.0 (5.6)
Total dairy (servings/day)	2.0 (1.3)	1.5 (0.4)	−0.5 (1.3)
Nuts (servings/day)	0.3 (0.5)	0.4 (0.8)	0.1 (0.9)
Yogurt (servings/day)	0.3 (0.5)	0.2 (0.3)	−0.1 (0.5)
White bread (servings/day)	0.4 (0.7)	0.2 (0.4)	−0.2 (0.7)
Tortillas (servings/day)	3.0 (1.9)	3.0 (1.8)	−0.0 (1.9)
Orange juice (servings/day)	0.4 (0.6)	0.3 (0.5)	−0.1 (0.7)

Age, soft drink intake, weight, physical activity (min/day and METS min/day), total daily sleep and food groups are reported as means and standard deviations. The other variables are reported as column percentages. Soft drink intake considered cola and flavor soft drink. A serving of soft drink represents a bottle of 355 ml. Chronic diseases include cirrhosis, diabetes, cardiovascular diseases, cancer and kidney diseases. Low physical activity category (< 21.44 min /day) is equal to < 150 min/week and high physical activity category (≥21.44 min /day) is equal to ≥150 min/week, according with physical activity recommendations of WHO

In model 1, after adjusting for demographic, lifestyle factors, diet, and physical activity, we found that an increase in soft drink intake of 1 serving per day was associated with weight gain of 0.10 kg (95% CI 0.00, 0.19) per year. In model 2, with the three-way interaction term, we found that the beta coefficient for the interaction between soft drink, physical activity and time was almost zero (β − 0.03 kg; 95% CI -0.27, 0.21). This suggests that the change in weight over time associated with soft drink intake does not differ by levels of physical activity. The best goodness of fit for the data was provided by the first model according to the AIC and BIC (Table 2).

**Table 2:** Association between soft drink intake and body weight change in the health workers cohort study, 2004–2010 (N = 1268)

Characteristics	Model 1 β-coefficient (95% CI)	Model 2 β-coefficient (95% CI)
Soft drink (servings/day)	0.03 (−0.64, 0.70)	− 0.02 (− 0.84, 0.79)
Time (years)	0.46 (0.23, 0.68)	0.45 (0.22, 0.68)
Soft drink x time	0.10 (0.00, 0.19)	0.11 (−0.02, 0.23)
Physical activity		
Low (referent)	–	–
High	0.42 (−0.19, 1.03)	0.34 (−0.40, 1.08)
Physical activity x time	−0.11 (− 0.23, 0.01)	−0.10 (− 0.24, 0.05)
Soft drink x physical activity	–	0.18 (− 0.84, 1.20)
Sex x time	0.08 (−0.03, 0.18)	0.07 (− 0.03, 0.18)
Baseline age x time	−0.02 (− 0.02, − 0.02)	−0.02 (− 0.02, − 0.02)
Soft drink x physical activity x time	–	− 0.03 (− 0.27, 0.21)
AIC test	12,149.59	12,153.29
BIC test	12,301.39	12,316.77

Model 1: Individual-level fixed effects model of two-way interaction terms (soft drink and time, physical activity and time, sex and time, and baseline age and time), adjusted for education, chronic diseases, smoking status, TV viewing time per week, total daily sleep, alcohol intake and food groups: red meat, total dairy, fruits, vegetables, nuts, yogurt, white bread, tortillas and orange juice. Model 2: Individual-level fixed effects model of three-way interaction terms (soft drink, time and physical activity), included two-way interaction terms (soft drink and time, physical activity and time, physical activity and soft drink, sex and time, and baseline age and time) adjusted by the same set of covariates of model 1. Age and sex were centered at the baseline mean in both models. *CI* confidence interval, *AIC* Akaike information criterion, *BIC* Bayesian information criterion

**Fig. 1:**
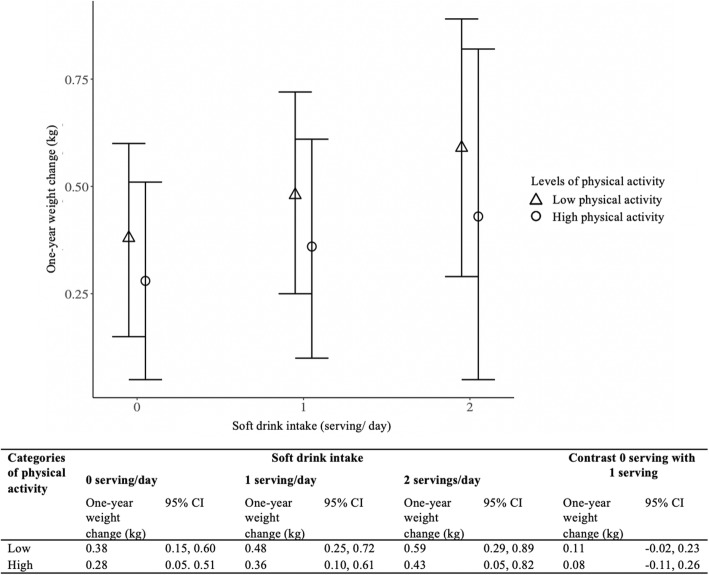
Annualized body weight change associated with soft drink intake by levels of leisure-time physical activity of three-way interaction model, 2004–2010 HWCS (N = 1268). CI: Confidence interval

Figure 1 shows the weight gain associated with soft drink intake for participants categorized “low” vs. those categorized “high” leisure-time physical activity. These are the predicted average marginal weight change estimates from model 2, that includes the non-significant triple interaction term. Among non-consumers of soft drink, people in the low physical activity category gained 0.38 kg/year, compared to 0.28 kg/year in the physically active participants (0.10 kg/year difference; 95% CI -0.05, 0.24 kg). Among people who consumed one serving of soft drink per day, those in the low physical activity category gained 0.48 kg/year, compared to 0.36 kg/year in the high physical activity category (0.13 kg/year difference, 95% CI -0.05, 0.31 kg). While the difference was not statistically significant, the estimate for the association of soft drink intake with weight change was smaller for individuals with higher leisure time physical activity than those with low leisure time physical activity. However, the focus of this analysis is to identify if the association between weight gain and soft drink intake varies by physical activity; contrasting the weight gain association with a one soft drink serving increase among those in the low physical activity group (0.11 kg/yr) versus those in the high physical activity group (0.08 kg/yr); the similarity between these two coefficients explains the lack of significance in the three-way interaction (see Table 2, model 2).

The sensitivity analyses consistently showed that, regardless of how we model leisure-time physical activity, the association between soft drink intake and weight gain persisted and was similar across levels of leisure-time physical activity. We calculated the predicted average weight change at different percentiles of physical activity and servings of soft drink, using physical activity as a continuous variable (minutes/day). The results were similar to model 2; even subjects who engaged in 60 min/day of physical activity gained weight, and weight gain still increased with higher levels of soft drink intake (see Table S1 and Fig. S3, available in the Additional file 2). Similar results were observed when we used METs min/day (see Table S2, available in the Additional file 2). Analyses stratified by baseline BMI showed similar results (see Table S3 and Table S4, available in Additional file 2).

## Discussion

The first aim was to estimate the association between changes in soft drink intake and weight change in a cohort of Mexican adults, with objective measurements of weight. We found that the soft drink intake was associated with weight gain, with an average of 0.10 kg/year/serving. The second aim assessed if the association between soft drink and weight differed by levels of physical activity. The results show that the amount of weight gain associated with soft drink intake was similar in people who engaged in low and high levels of leisure-time physical activity. In other words, leisure-time physical activity did not modify the association of weight gain with soft drink intake in this sample.

Meta-analytical evidence has consistently shown that SSB intake is associated with weight gain in adults, after adjustment for physical activity [1, 19, 20, 34]. However, these studies have been mostly conducted in developed countries and used baseline SSB intake to estimate weight gain, with no consideration for SSB intake changes over follow-up. In this study, a 1-serving increase in daily soft drink intake was associated with an additional 0.10 kg annual weight gain, after adjustment for leisure-time physical activity and other covariates. The result of this analysis is very similar to the result of the meta-analysis by Malik, et al., which used a similar modeling approach (0.12 kg/year, 95% CI: 0.10, 0.14) [19]. Nevertheless, this meta-analysis did not include evidence from Hispanic populations. A study conducted in Mexican women found an increase of 1.0 kg (95% CI 0.7, 1.2) per soft drink serving over 2 years of follow-up, suggesting a stronger association than estimated by this analysis [15]. This difference could be due to the use of self-reported weight, compared to the objective weight assessment from this study. There are also important differences in the participants of each cohort study; in this case, it is mostly constituted by men and women working in a healthcare public system, while Stern’s, et al., is composed of female teachers.

This study also provides further evidence that high levels of physical activity do not modify the weight gain associated with soft drink intake. According to the statistical model, subjects who meet the WHO physical activity guidelines did not reach the amounts of physical activity necessary to counteract the energy consumed from soft drink. In line with these results, a prospective study among adolescents and adults found that vigorous physical activity did not modify the association between sports drinks intake and weight gain [35]. This evidence challenges the idea that the calories burned with leisure-time physical activity can offset the caloric intake of SSB [36–38]. Furthermore, there was no significant difference in the weight gained by each SSB serving, even at high levels of physical activity (PA) (60 min/day). We suggest that the explanation for this observation is related to the complex regulation of calories by solids and liquids. Theoretically, all caloric intake could be offset by engaging in sufficient physical activity [39, 40]. However, liquid calories are known to produce less appetite suppression than solids and lower energy compensation [41–46]. Thus, caloric regulation through appetite is mostly based on solids and not liquids. This could explain the similar weight gain associated with soft drink intake across levels of PA: people should be adjusting their caloric intake based on solids, making every soft drink serving an extra caloric source that is reflected in weight gain. Further experimental studies on the caloric dynamics of SSB intake are needed to better understand their role in weight gain.

According to the statistical model it is implausible to balance weight gain only by increasing physical activity levels at the population-level. Physical activity needs to match the intake of solid food and liquids in a given population to achieve energy balance. In modern societies, only a small proportion of the population engages in sufficiently high levels of physical activity to balance out the caloric intake from highly affordable and calorie-dense foods [47]. To put the results in context, 72.3% of the Mexican population complies with the WHO recommendations for physical activity (75 min/week of vigorous physical activity or 150 min/week of moderate physical activity) [28, 48]. Yet, according to the National Health and Nutrition Survey 2012, the mean caloric intake from soda was 207 kcal/day among adults aged ≥20 years old, equivalent to drinking 1.4 servings of soft drink/day. To offset this caloric intake, without taking into consideration other caloric sources, an average 70 kg adult would need to perform 588 min/week of vigorous physical activity (WHO recommends 210 min/week) or 662 min/week of moderate physical activity (WHO recommends 315 min/week) [49]. While some individuals may be healthy and able to reach the aforementioned physical activity level, achieving population weight control only through physical activity was implausible in the individuals of the sample. Physical activity is an integral part of a healthy lifestyle, and plenty of evidence supports its promotion to provide health benefits for weight gain [50]. However, diet is the major contributor of weight gain at the population level. In fact, the Mexican diet is increasingly comprised of ultra-processed foods, which has been shown to be highly palatable and linked with excessive weight gain [51–54].

Some limitations must be considered. Measurement error from self-reported questionnaires such as the FFQ and physical activity needs to be acknowledged. Soft drink intake measured using an FFQ may differ from usual intake, leading to misclassification bias. Yet, for that misclassification bias to explain these findings, we would need for recall to induce higher reporting in people who gained weight and lower reporting amongst people who did not gain weight. Weight trajectories are heavily influenced by baseline values. Thus, to explore this possibility, we stratified the models by baseline weight. Both, people with and without overweight and obesity, showed similar results (see Table S3 and Table S4, available in Additional file 2). This suggests that these findings are not linked to obesity status at baseline or other variables related to it, such as soft drink recall due to obesity or weight gain. Likewise, levels of physical activity can vary according to BMI categories. However, the distribution of low and high levels of leisure-time physical activity was similar in people with obesity, overweight and normal/underweight weight. Overall, the literature suggests, if anything, that overweight people underestimate their caloric intake, particularly on perceived unhealthy foods and beverages [55–58]. This would lead to an underestimate of SSB intake among overweight individuals and lead to a larger physical activity effect. This cohort study is relatively small, which could limit the ability to detect statistical significance of the three-way interaction. Yet, the coefficient of the triple interaction was near zero, with both positive and negative effects being equally likely. Thus, it is implausible that increasing the sample size would lead to a clinically relevant effect modification of leisure-time physical activity in the association between soft drink and weight gain. Given the observational nature of this study, we cannot rule out the possibility of residual confounding. Finally, this cohort is not a representative sample of Mexican adults due to different prevalence of chronic diseases with the general population level. Despite these potential limitations, the study has several strengths. We used a prospective study design to analyze a direct path between soft drink intake and weight change. We used objective measurements for weight. We also used an analytical approach that controls for time-invariant confounders, whether they were measured or not. Additionally, the statistical models were controlled by covariates of food groups linked with soft drink intake and weight change. The results were robust for the different sensitivity analyses. Sociodemographic characteristics were similar between the analytic and the full sample, yet, out analytical sample had slightly less women and more participants with secondary and high-school education (see Table S5, available in Additional file 2). Finally, these results can be generalized to other regions where the average soft drink intake is similar to the one of the HWCS population if the modifiers of the association are distributed equally.

## Conclusion

In a cohort of Mexican adults, we found that soft drink intake was associated with weight gain. We failed to observe a modifying effect of physical activity over the soft drink intake and weight association; physical activity levels would need to be much higher than observed in this cohort to counterbalance excess energy intake. Public health policies should focus on the regulation of SSB intake at population level, beyond promoting physical activity.

## Supplementary information

**Additional file 1: Figure S1.** Directed Acyclic Graph (DAG) of the association of soft drink intake and weight change, with the effect modification by physical activity. Figure about the association between soft drink intake, body weight, and physical activity. **Figure S2.** Participants flow diagram of the Health Workers Cohort Study, 2004–2010. Figure containing the flowchart of the exclusion criteria of the sample from the Health Workers Cohort Study. Statistical models. File explaining the statistical models used in the analysis.

**Additional file 2: Table S1.** Association between soft drink intake and body weight change using physical activity in minutes per day (continuous variable), in the Health Workers Cohort Study, 2004–2010 (*N* = 1268). Table containing the results of the fixed effect model with a three-way interaction term between soft drink, time, and leisure-time physical activity in minutes per day (continuous variable). **Figure S3.** Annualized body weight change associated with soft drink intake by levels of leisure-time physical activity (continuous variable) of three-way interaction model, 2004–2010 HWCS (*N* = 1268). Figure containing the predicted average marginal weight change estimates from fixed effect model with a three-way interaction term between soft drink, time, and leisure-time physical activity in minutes per day (continuous variable). **Table S2.** Association between soft drink intake and body weight change using metabolic equivalent units (METS) minutes per day, in the Health Workers Cohort Study, 2004–2010 (*N* = 1268). Table containing the results of the fixed effect model with a three-way interaction term between soft drink, time, and leisure-time physical activity in METS min/day (categorical variable). **Table S3.** Annualized body weight change associated with soft drink intake by levels of leisure-time physical, in people with BMI < 25 kg/m^2^, 2004–2010 HWCS (*N* = 506). Table containing the predicted average marginal weight change estimates from fixed effect model with a three-way interaction term between soft drink, time, and leisure-time physical activity, in people with BMI < 25 kg/m^2^. **Table S4.** Annualized body weight change associated with soft drink intake by levels of leisure-time physical, in people with BMI ≥25 kg/m^2^, 2004–2010 HWCS (*N* = 762). Table containing the predicted average marginal weight change estimates from fixed effect model with a three-way interaction term between soft drink, time, and leisure-time physical activity, in people with BMI ≥25 kg/m^2^. **Table S5.** Sociodemographic characteristics in full and analytic samples for 2004. Table containing differences in the sociodemographic characteristics between full sample and analytical sample at baseline.

## Data Availability

The cohort data supporting the conclusions of this article are not publicly available. The material supporting the conclusions of this article are included in additional files.

## Abbreviations

WHO: World Health Organization
SSB: Sugar sweetened beverages
HWCS: Health workers cohort study
FFQ: Food frequency questionnaire
AIC: Akaike Information Criterion
BIC: Bayesian Information Criterion
METs: Minutes of Metabolic Equivalent units
