# Supplementary material for: Soft drink intake is associated with weight gain, regardless of physical activity levels: the health workers cohort study
Source: Int J Behav Nutr Phys Act. 2020 May 12;17:60. doi: 10.1186/s12966-020-00963-2 (PMC7216416; doi:10.1186/s12966-020-00963-2)
Supplement: Supplementary file 1 — Additional file 1: Figure S1. Directed Acyclic Graph (DAG) of the association of soft drink intake and weight change, with the effect modification by physical activity. Figure about the association between soft drink intake, body weight, and physical activity. Figure S2. Participants flow diagram of the Health Workers Cohort Study, 2004–2010. Figure containing the flowchart of the exclusion criteria of the sample from the Health Workers Cohort Study. Statistical models. File explaining the statistical models used in the analysis. [file 12966_2020_963_MOESM1_ESM.docx]

**ADDITIONAL FILE 1**

**Figure S1. Directed Acyclic Graph (DAG) of the association of soft drink intake and weight change, with the effect modification by physical activity.**

**
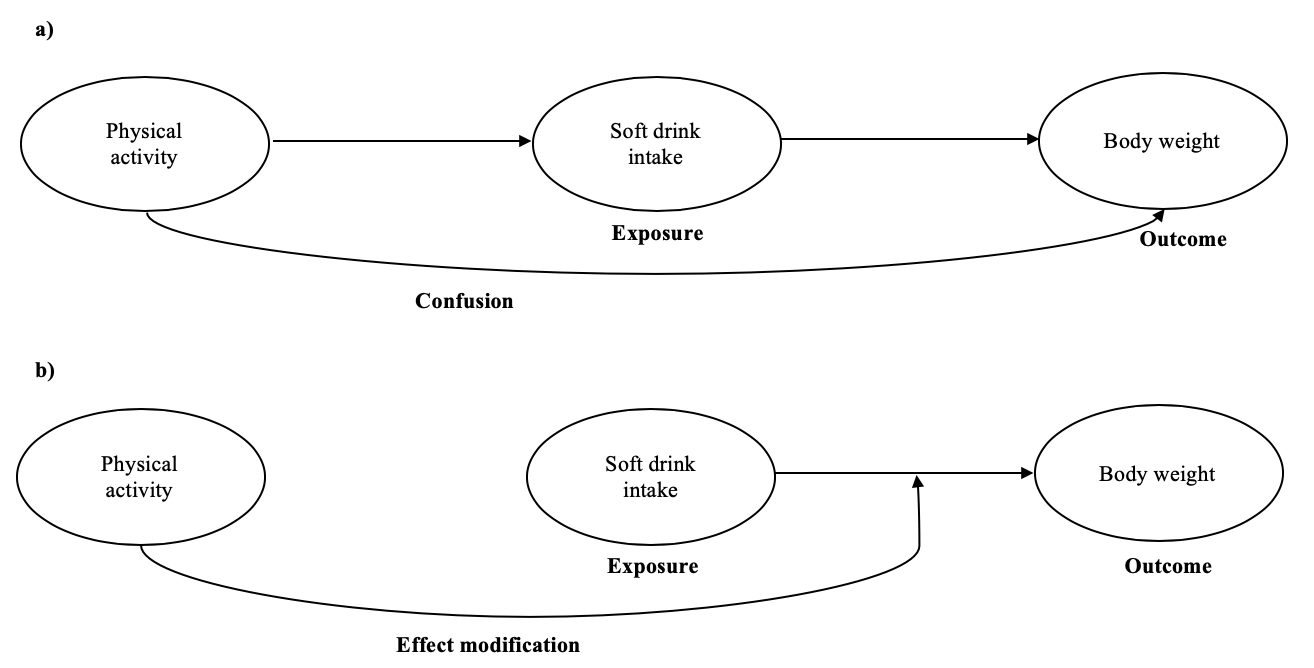
**

DAG with physical activity variable like a confounder (a) and an effect modifier (b).

**Figure S2. Participants flow diagram of the Health Workers Cohort Study, 2004-2010.**

**
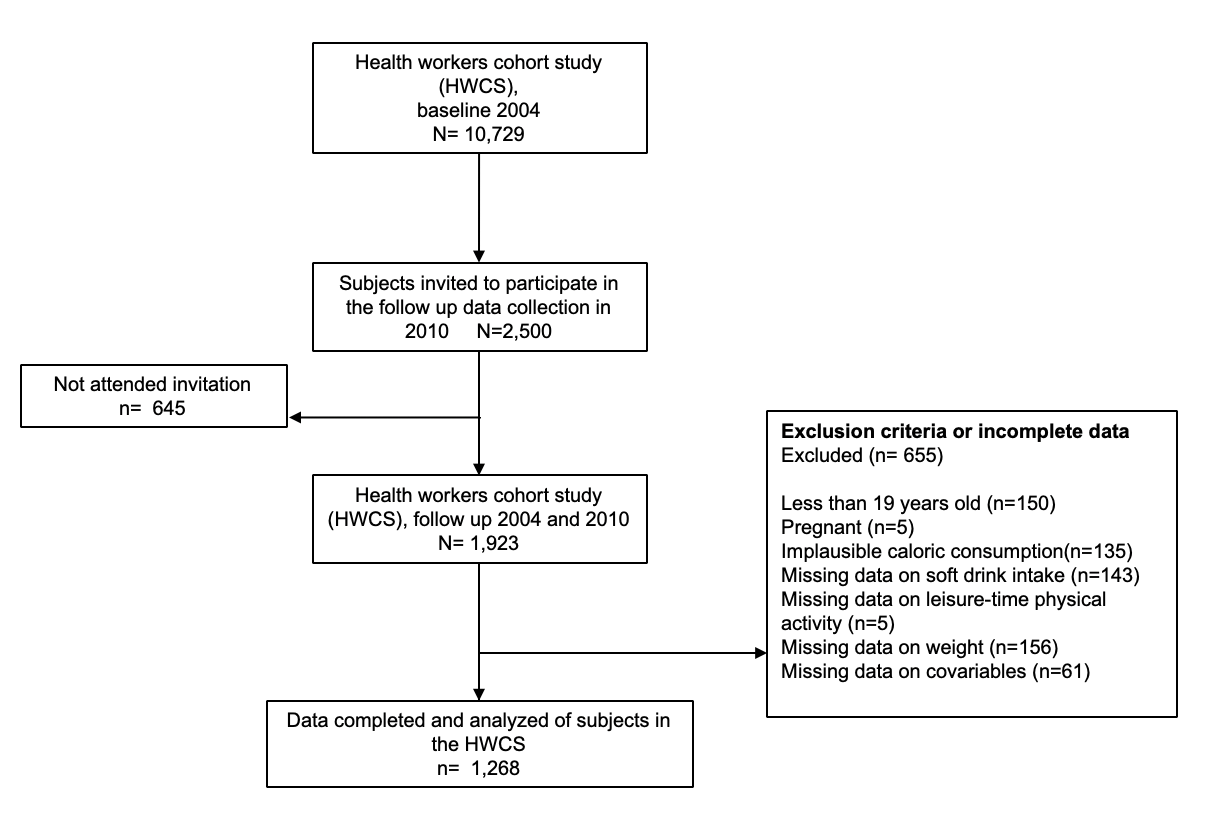
**

**Statistical models**

**Model 1**

$Y= {}_{0}+ {}_{1}\left( {Time}_{it} \right)+{}_{2}\left( {Soft drink}_{it} \right)+{}_{3}\left( {Physical activity}_{it} \right)+{}_{4}\left( {Soft drink}_{it} x {Time}_{it} \right)+{}_{5}\left( {Physical activity}_{it} x {Time}_{it} \right)+{}_{6}\left( {Sex}_{it} x {Time}_{it} \right)+{}_{7}\left( {Baseline age}_{it} x {Time}_{it} \right)+$ ${}_{n}({Covariates}_{it})$ $+$ $u_{it}$

${}_{0}$= Intercept

${}_{1}\left( {Time}_{it} \right)$= Time when soft drink intake is 0, adjusting for physical activity and covariates for subject *i* in the time *t*

${}_{2}\left( {Soft drink}_{it} \right)$= Soft drink intake when time is 0, adjusting for physical activity and covariates for subject *i* in the time *t*

${}_{3}\left( {Physical activity}_{it} \right)=$ Physical activity when time is 0, adjusting for covariates for subject *i* in the time *t*

${}_{4}\left( {Soft drink}_{it} x {Time}_{it} \right)$= Yearly trajectory of soft drink intake for subject *i* in the time *t*

${}_{5}\left( {Physical activity}_{it} x {Time}_{it} \right)$= Yearly trajectory of physical activity for subject *i* in the time *t*

${}_{6}\left( {Sex}_{it} x {Time}_{it} \right)$= Yearly trajectory of weight according sex for subject *i* in the time *t*

${}_{7}\left( {Baseline age}_{it} x {Time}_{it} \right)$= Yearly trajectory of weight according baseline age for subject *i* in the time *t*

${}_{n}({Covariates}_{it})$ = Education, smoking status, screen-time per week, sleep, alcohol intake, chronic diseases and food groups.

$u_{it}$= Error term

**Model 2**

$Y= {}_{0}+ {}_{1}\left( {Time}_{it} \right)+{}_{2}\left( {Soft drink}_{it} \right)+{}_{3}\left( {Physical activity}_{it} \right)+{}_{4}\left( {Soft drink}_{it} x {Time}_{it} \right)+{}_{5}\left( {Physical activity}_{it} x {Time}_{it} \right)+ +{}_{6}\left( {Soft drink}_{it} x {Physical activity}_{it} \right) +{}_{7}\left( {Soft drink}_{it} x{Physical activity}_{it} x {Time}_{it} \right)+{}_{8}\left( {Sex}_{it} x {Time}_{it} \right)+{}_{9}\left( {Baseline age}_{it} x {Time}_{it} \right)+$ ${}_{n}({Covariates}_{it})$ $+$ $u_{it}$

${}_{0}$= Intercept

${}_{1}\left( {Time}_{it} \right)$= Time when soft drink intake is 0, adjusting for physical activity and covariates for subject *i* in the time *t*

${}_{2}\left( {Soft drink}_{it} \right)$= Soft drink intake when time is 0, adjusting for physical activity and covariates for subject *i* in the time *t*

${}_{3}\left( {Physical activity}_{it} \right)=$ Physical activity when time is 0, adjusting for covariates for subject *i* in the time *t*

${}_{4}\left( {Soft drink}_{it} x {Time}_{it} \right)$= Yearly trajectory of soft drink intake, adjusting for physical activity for subject *i* in the time *t*

${}_{5}\left( {Physical activity}_{it} x {Time}_{it} \right)$= Yearly trajectory of physical activity for subject *i* in the time *t*

${}_{6}\left( {Soft drink}_{it} x {Physical activity}_{it} \right)$= Soft drink intake when time is 0 and physical activity is 1, adjusting for covariates for subject *i* in the time *t*

${}_{7}\left( {Soft drink}_{it} x {Physical activity}_{it} x {Time}_{it} \right)$= Soft drink intake when physical activity is 1 and time is 1, adjusting for covariates for subject *i* in the time *t*

${}_{n}({Covariates}_{it})$ = Education, smoking status, screen-time per week, sleep, alcohol intake, chronic diseases and food groups.

$u_{it}$= Error term
