# Supplementary material for: Soft drink intake is associated with weight gain, regardless of physical activity levels: the health workers cohort study
Source: Int J Behav Nutr Phys Act. 2020 May 12;17:60. doi: 10.1186/s12966-020-00963-2 (PMC7216416; doi:10.1186/s12966-020-00963-2)
Supplement: Supplementary file 2 — Additional file 2: Table S1. Association between soft drink intake and body weight change using physical activity in minutes per day (continuous variable), in the Health Workers Cohort Study, 2004–2010 (N = 1268). Table containing the results of the fixed effect model with a three-way interaction term between soft drink, time, and leisure-time physical activity in minutes per day (continuous variable). Figure S3. Annualized body weight change associated with soft drink intake by levels of leisure-time physical activity (continuous variable) of three-way interaction model, 2004–2010 HWCS (N = 1268). Figure containing the predicted average marginal weight change estimates from fixed effect model with a three-way interaction term between soft drink, time, and leisure-time physical activity in minutes per day (continuous variable). Table S2. Association between soft drink intake and body weight change using metabolic equivalent units (METS) minutes per day, in the Health Workers Cohort Study, 2004–2010 (N = 1268). Table containing the results of the fixed effect model with a three-way interaction term between soft drink, time, and leisure-time physical activity in METS min/day (categorical variable). Table S3. Annualized body weight change associated with soft drink intake by levels of leisure-time physical, in people with BMI < 25 kg/m2, 2004–2010 HWCS (N = 506). Table containing the predicted average marginal weight change estimates from fixed effect model with a three-way interaction term between soft drink, time, and leisure-time physical activity, in people with BMI < 25 kg/m2. Table S4. Annualized body weight change associated with soft drink intake by levels of leisure-time physical, in people with BMI ≥25 kg/m2, 2004–2010 HWCS (N = 762). Table containing the predicted average marginal weight change estimates from fixed effect model with a three-way interaction term between soft drink, time, and leisure-time physical activity, in people with BMI ≥25 kg/m2. Table [file 12966_2020_963_MOESM2_ESM.docx]

**ADDITIONAL FILE 2**

**Table S1. Association between soft drink intake and body weight change using physical activity in minutes per day (continuous variable), in the Health Workers Cohort Study, 2004-2010 (N=1 268).**

| **Characteristics** | **Model** |
| --- | --- |
|  | b-coefficient (95% CI) |
| Soft drink (servings/day) | -0.09 (-0.71, 0.52) |
| Time (years) | 0.46 (0.22, 0.69) |
| Soft drink x time | 0.11 (0.01, 0.22) |
| Physical activity | 0.00 (-0.01, 0.02) |
| Physical activity x time | -0.00 (-0.01, 0.00) |
| Soft drink x physical activity | 0.01 (-0.01, 0.02) |
| Sex x time | 0.07 (-0.03, 0.18) |
| Baseline age x time | -0.02 (-0.03, -0.02) |
| Soft drink x physical activity x time | -0.00 (-0.00, 0.00) |

Model: Individual-level fixed effects model of three-way interaction terms (soft drink x time x continuous variable of physical activity), adjusted for two-way interaction terms (sex x time, baseline age x time), education, chronic diseases, smoking status, TV viewing time per week, total daily sleep, alcohol intake and food groups: red meat, total dairy, fruits, vegetables, nuts, yogurt, white bread, tortillas and orange juice. Age and sex were centered at the baseline mean. CI: confidence interval

**Figure S3. Annualized body weight change associated with soft drink intake by levels of leisure-time physical activity (continuous variable) of three-way interaction model, 2004-2010 HWCS (N=1 268).**

**
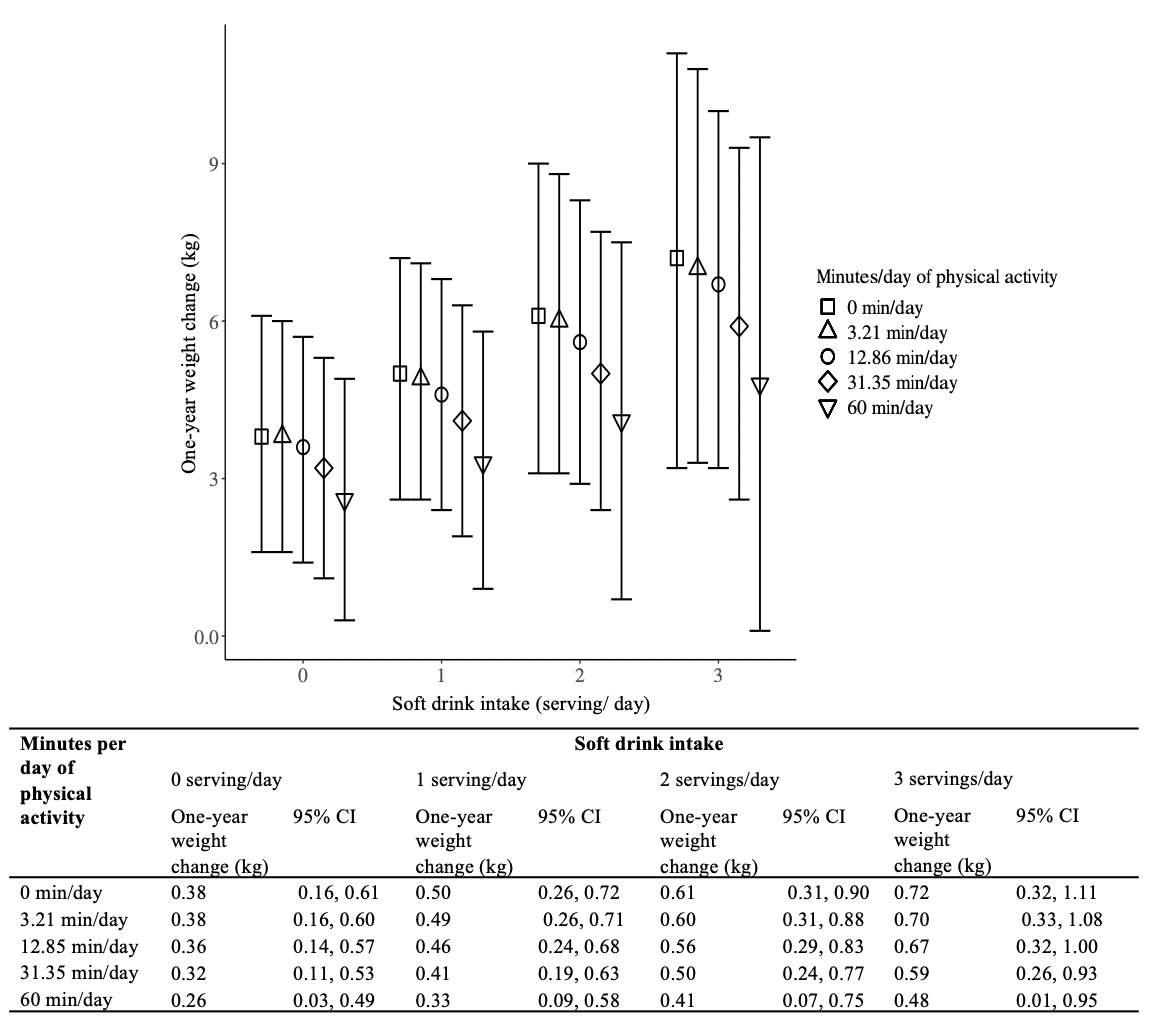
**

CI: Confidence interval

**Table S2. Association between soft drink intake and body weight change using metabolic equivalent units (METS) minutes per day, in the Health Workers Cohort Study, 2004-2010 (N=1 268).**

| **Characteristics** | **Mode**l |
| --- | --- |
|  | b-coefficient (95% CI) |
| Soft drink (servings/day) | 0.03 (-0.61, 0.68) |
| Time (years) | 0.42 (0.20, 0.63) |
| Soft drink x time | 0.10 (0.00, 0.20) |
| Physical activity (METs min/day) |  |
| Low (referent) | - |
| High | 0.21 (-0.54, 0.95) |
| Physical activity x time | -0.09 (-0.23, 0.05) |
| Soft drink x physical activity | 0.03 (-1.03, 1.09) |
| Sex x time | 0.07 (-0.04, 0.18) |
| Baseline age x time | -0.02 (-0.03, -0.02) |
| Soft drink x physical activity x time | -0.01 (-0.22, 0.25) |

Model: Individual-level fixed effects model of three-way interaction terms (soft drink x time x physical activity in METS), adjusted for two-way interaction terms (sex x time, baseline age x time), education, chronic diseases, smoking status, TV viewing time per week, total daily sleep, alcohol intake and food groups: red meat, total dairy, fruits, vegetables, nuts, yogurt, white bread, tortillas and orange juice. Age and sex were centered at the baseline mean. CI: confidence interval.

**Table S3. Association between soft drink intake and body weight change in people with BMI <25kg/m^2^, in the Health Workers Cohort Study, 2004-2010 (N=506).**

| **Characteristics** | **Model 1** | **Model 2** |
| --- | --- | --- |
|  | β-coefficient (95% CI) | β-coefficient (95% CI) |
| Soft drink (servings/day) | 0.45 (-0.34, 1.24) | 0.47 (-0.49, 1.43) |
| Time (years) | 0.53 (0.16, 0.89) | 0.53 (0.17, 0.90) |
| Soft drink x time | 0.09 (-0.03, 0.22) | 0.08 (0.08, 0.24) |
| Physical activity |  |  |
| Low (referent) | - | - |
| High | 0.77 (-0.08, 1.61) | 0.80 (-0.21, 1.82) |
| Physical activity x time | -0.14 (-0.30, 0.02) | -0.16 (-0.36, 0.04) |
| Soft drink x physical activity | - | -0.05 (-1.44, 1.34) |
| Sex x time | 0.07 (-0.08, 0.22) | 0.07 (-0.08, 0.22) |
| Baseline age x time | -0.02 (-0.03, -0.02) | -0.02 (-0.03, -0.02) |
| Soft drink x physical activity x time | - | -0.06 (-0.26, 0.37) |
| AIC test | 4495.82 | 4499.49 |
| BIC test | 4628.65 | 4642.16 |

Model 1: Individual-level fixed effects model of two-way interaction terms (soft drink and time, physical activity and time, sex and time, and baseline age and time), adjusted for education, chronic diseases, smoking status, TV viewing time per week, total daily sleep, alcohol intake and food groups: red meat, total dairy, fruits, vegetables, nuts, yogurt, white bread, tortillas and orange juice. Model 2: Individual-level fixed effects model of three-way interaction terms (soft drink, time and physical activity), included two-way interaction terms (soft drink and time, physical activity and time, physical activity and soft drink, sex and time, and baseline age and time) adjusted by the same set of covariates of model 1. Age and sex were centered at the baseline mean in both models. CI: confidence interval. AIC: Akaike information criterion. BIC: Bayesian information criterion.

**Table S4. Association between soft drink intake and body weight change in people with BMI ≥25kg/m^2^, in the Health Workers Cohort Study, 2004-2010 (N=762).**

| **Characteristics** | **Model 1** | **Model 2** |
| --- | --- | --- |
|  | β-coefficient (95% CI) | β-coefficient (95% CI) |
| Soft drink (servings/day) | -0.24 (-0.94, 0.46) | -0.31 (-1.09, 0.47) |
| Time (years) | 0.44 (0.13, 0.74) | 0.43 (0.12, 0.74) |
| Soft drink x time | 0.08 (-0.04, 0.20) | 0.10 (-0.04, 0.24) |
| Physical activity |  |  |
| Low (referent) | - | - |
| High | 0.09 (-0.79, 0.97) | 0.03 (-1.09, 1.03) |
| Physical activity x time | -0.08 (-0.25, 0.08) | -0.06 (-0.27, 0.15) |
| Soft drink x physical activity | - | 0.25 (-1.02, 1.52) |
| Sex x time | 0.05 (-0.10, 0.19) | 0.05 (-0.10, 0.19) |
| Baseline age x time | -0.02 (-0.02, -0.12) | -0.02 (-0.02, -0.02) |
| Soft drink x physical activity x time | - | -0.07 (-0.38, 0.24) |
| AIC test | 7555.61 | 7559.15 |
| BIC test | 7699.50 | 7713.70 |

Model 1: Individual-level fixed effects model of two-way interaction terms (soft drink and time, physical activity and time, sex and time, and baseline age and time), adjusted for education, chronic diseases, smoking status, TV viewing time per week, total daily sleep, alcohol intake and food groups: red meat, total dairy, fruits, vegetables, nuts, yogurt, white bread, tortillas and orange juice. Model 2: Individual-level fixed effects model of three-way interaction terms (soft drink, time and physical activity), included two-way interaction terms (soft drink and time, physical activity and time, physical activity and soft drink, sex and time, and baseline age and time) adjusted by the same set of covariates of model 1. Age and sex were centered at the baseline mean in both models. CI: confidence interval. AIC: Akaike information criterion. BIC: Bayesian information criterion.

**Table S5. Sociodemographic characteristics in full and analytic samples for 2004.**

| **Sociodemographic characteristics** | **Full sample** | **Analytical sample** | **P value** |
| --- | --- | --- | --- |
|  | n=1942 | n= 1268 |  |
|  | Mean (SD) | Mean (SD) |  |
| Sex, % |  |  |  |
| Women | 76.1 | 74.2 | 0.01 |
| Age (years) | 45.2 (12.9) | 45.3 (12.7) | 0.68 |
| Education, % |  |  |  |
| Elementary school | 12.7 | 12.4 | 0.10 |
| Secondary or high school | 35.8 | 37.4 |  |
| College or higher | 51.5 | 50.2 |  |

SD: Standard deviation
